# The first complete mitogenome of *Onychostoma ovale* (Pellegrin & Chevey, 1936) with phylogenetic analyses

**DOI:** 10.1080/23802359.2022.2119819

**Published:** 2022-09-15

**Authors:** Congqiang Luo, Zemin Ma, Ping Chen, Pinhong Yang

**Affiliations:** a Changde Innovation Team of Wetland Biology and Environmental Ecology, Changde, China; b Hunan Provincial Key Laboratory for Molecular Immunity Technology of Aquatic Animal Diseases, Changde, China; c Changde Dingcheng District Animal Husbandry and Fishery Affairs Center, Changde, China

**Keywords:** *Onychostoma ovale*, mitogenome, phylogenetic status

## Abstract

*Onychostoma ovale* (Pellegrin & Chevey, 1936) is an endemic cyprinid fish that is widely inhabited in southern China, Vietnam, and Laos. In the present study, we first reported the complete mitochondrial genome of *O. ovale*. The mitogenome contained 16,600 bp with AT content of 56.2% and comprised of 13 protein-coding genes, 22 transfer RNA genes, two ribosomal RNA genes, and one control region (D-loop). Phylogenetic analyses suggested that *Onychostoma* species formed two major clades and the subspecies of *O. ovale* had close relationship with *O. rarum*. The mitochondrial genome of *O. ovale* provided a key aid for population genetics and phylogenetic inferences of *Onychostoma* in future research.

*Onychostoma* is an important fish group in Cyprinidae that contains 23 valid species (www.fishbase.org). *Onychostoma ovale* (Pellegrin & Chevey, 1936) is a representative member in *Onychostoma* that is widely distributed in the Southern China, Vietnam, and Laos (Yue 2000; www.fishbase.org). Though this species has a wide distribution range, the species was not seen in recent field sampling in Southern China due to rare resources (Li et al. 2012; Zhang et al. 2020; Zhu et al. 2022), suggesting that more attention should be paid for this subspecies. To date, nevertheless, the information of this species is pretty poor as sparse researches have been conducted researches for this species, especially genetic studies. In this study, we first sequenced the whole mitochondrial genome of *O. ovale* and tried to resolve its phylogenetic status.

*Onychostoma ovale* specimen was captured from local fishermen in a small local market of Du’an County, Guangxi Province, China (23.952N, 108.089E) on 27 July 2021. We diagnosed this sample according to the fish fauna by Yue (2000). Total genomic DNA was extracted from muscle tissue using a Genomic DNA Isolation Kit (QiaGene, Hilden, Germany). The sample and the total DNA were preserved in the fish collection of Hunan University of Arts and Science (www.huas.edu.cn, Zemin Ma and Flysu210610@163.com) under the voucher numbers LXBJ20210701. The complete mitogenome of *O. ovale* was obtained via sequencing the DNA using the Illumina MiSeq platform (Illumina Inc., San Diego, CA) and assembled by SPAdes 3.9.0 (Bankevich et al. 2012). The assembled mitochondrial genomes were annotated using the online tool MitoAnnotator (http://mitofish.aori.u-tokyo.ac.jp/annotation; Iwasaki et al. 2013). The annotated mitogenome was deposited in GenBank with the accession number ON120245.

The complete mitogenome of *O. ovale* reached 16,600 bp in length with the A + T content (55.6%) was much higher than its G + C content (44.4%). It comprised of 13 protein-coding genes, two rRNA genes (12S rRNA and 16S rRNA), 22 tRNA genes, and a control region (D-loop), as observed in other fish species (Mascolo et al. 2018; Zhai et al. 2020). The initiation codon for all 13 PCGs was ATG except COI where it was GTG. With regard to the stop codon, six protein-coding genes (ND1, COI, ATP8, ND4L, ND5, and ND6) performed the routine termination codon (TAA or TAG), whereas five other protein-coding genes (ND2, COII, ND3, ND4, and Cytb) stopped with an incomplete stop codon T and two protein-coding genes (COIII and ATP6) stopped with an incomplete stop codon TA.

In order to understand the phylogenetic status of *O. ovale,* we downloaded 11 published *Onychostoma* mitogenomes and constructed Bayesian inference (BI) and maximum-likelihood (ML) trees using the concatenated supergene that contained 13 protein-coding genes. The ML and BI trees were built using RAXML-VI-HPC (Stamatakis 2006) and MrBayes 3.2 (Ronquist and Huelsenbeck 2003), respectively. *Acrossocheilus beijiangensis* (GenBank no. KY131976) was selected as an outgroup. The best nucleotide substitution model of GTR + I+G was chosen using MRMODELTEST version 2.3 on the basis of the Akaike information criterion (Nylander 2004). Both trees consistently resolved two major clades, which was in line with a previous study by Zhai et al. (2020). In addition, this study indicated that *O. ovale* was closely related to *O. rarum* with high supported values (Figure 1). In future studies, the mitochondrial genome of *O. ovale* is helpful for phylogenetic inferences of *Onychostoma*. Furthermore, appropriate mitochondrial genes can be selected from *O. ovale* mitogenome to conduct conservation genetic studies of *O. ovale* populations.

**Figure 1.**
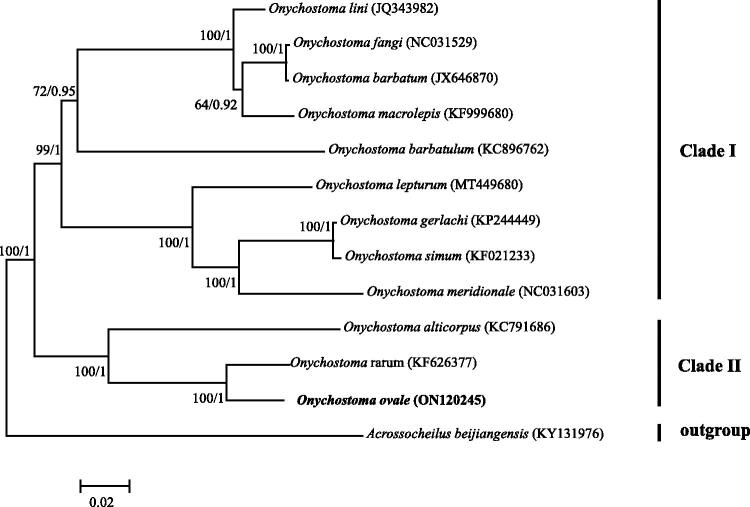
Maximum-likelihood tree showing the phylogenetic relationships among *Onychostoma* species using 13 protein-coding genes. Values on branches represent bootstrap values from maximum-likelihood analysis and posterior probability from Bayesian inference.

## Ethical approval

Experiments were performed in accordance with the recommendations of the Ethics Committee of Hunan University of Arts and Science. These policies were enacted according to the Chinese Association for the Laboratory Animal Sciences and the Institutional Animal Care and Use Committee (IACUC) protocols.

## Author contributions

Congqiang Luo and Zemin Ma: conception, design, analysis, investigation, interpretation of the data, and writing and revising the draft. Ping Chen: investigation. Pinhong Yang: design, investigation, and revising the draft. All authors agree to be accountable for all aspects of the work.

## Data Availability

The genome sequence data that support the findings of this study are openly available in GenBank of NCBI at https://www.ncbi.nlm.nih.gov/ under the accession no. ON120245. The associated BioProject, SRA, and Bio-Sample numbers of specimen are PRJNA821555, SRR18550323, and SAMN27115798, respectively.
